# Eutectic solvents with tuneable hydrophobicity: lipid dissolution and recovery†

**DOI:** 10.1039/d1ra00306b

**Published:** 2021-02-19

**Authors:** Calvin Lo, Jeltzlin Semerel, Corjan van den Berg, René H. Wijffels, Michel H. M. Eppink

**Affiliations:** Bioprocess Engineering, AlgaePARC, Wageningen University PO Box 16 6700 AA Wageningen The Netherlands calvin.lo@wur.nl +31 317485289; Faculty of Biosciences and Aquaculture, Nord University N-8049 Bodø Norway

## Abstract

Despite the promising advantages of eutectic solvents, the application of these solvents as an extraction solvent is still limited due to the challenging product recovery. Previously, it was reported that lipids could be recovered from a hydrophobic eutectic solvent with the principle of switchable hydrophobicity. However, this method still involves additional chemicals, such as polymeric amines, water, and CO_2_, which need to be removed later. In this study, we proposed a different approach by shifting the hydrophobicity spectrum of a semi-hydrophobic solvent. Made of hydrophilic imidazole and hydrophobic hexanoic acid, this combination showed tuneable hydrophobicity when the composition was changed, shown by the change of dipolarity (π*) scale from solvatochromic analysis. At low imidazole content, the solvent was able to dissolve sunflower oil and algae oil, whereas, at high imidazole content, the solvent showed high affinity towards water. By adding imidazole to the solution of oil and the solvent, a phase split was induced between the oil-rich upper phase and the solvent-rich lower phase. With this approach, ∼75% of recovery efficiency was achieved for the two oils, with the purity of ∼100% for sunflower oil and 86% for algae oil.

## Abstract 2

Depending on the composition, eutectic solvent imidazole/hexanoic acid has a tuneable hydrophobicity. In this study, this principle was used for lipid dissolution and recovery.

## Graphical abstract

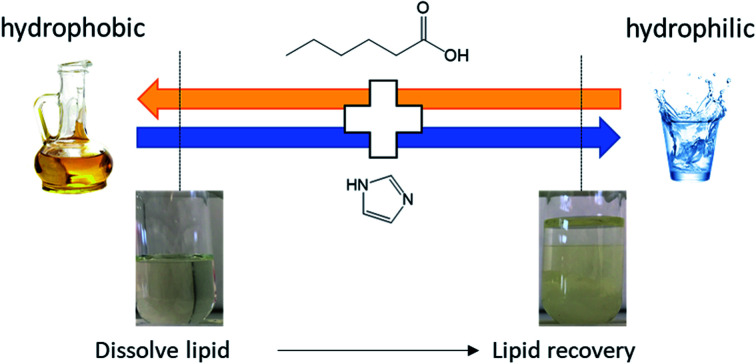

## Introduction

Eutectic solvents, including “deep eutectic solvents” (DES), have been gaining interest as alternative green solvents in the field of extraction of natural compounds. This is due to the attractive advantages of these solvents, such as low vapour pressure, high thermal stability, and high carrying capacity.^1–3^ Moreover, a eutectic solvent can be cheaply and easily prepared by mixing Brønsted or Lewis acid and base or “hydrogen bond donor” and “hydrogen bond acceptor”.^1,2,4,5^ Furthermore, the properties of these solvents are claimed to be designable.^6–8^ For example, if the mixture is made of bio-derived, safe, and biodegradable compounds, then the solvent might also be benign and sustainable.^5,9,10^

These mixtures melt at lower temperatures than the parental constituents, such that allows the mixtures to be liquid at room temperature, even if the parental compounds are solids.^11–15^ The depression of melting point can be found in not only “deep eutectic solvents”, but also normal, ideal mixtures. Group of Coutinho^13^ proposed to specifically classify DES as mixtures which exhibit further reduction of melting point when compared to ideal eutectic mixture. In this study, we use the term of eutectic solvents to have wider selection of mixtures, regardless the deviation of the melting point reduction.

Despite the advantages, the use of eutectic solvents still faces several challenges, such as difficult product recovery and solvent regeneration.^16^ This is mainly because these solvents have low volatility. In the typical organic solvent process, these two processes were done *via* distillation and solvent condensation. However, applying distillation to eutectic solvents would require a tremendous amount of energy. Therefore, other strategies need to be developed to tackle this bottleneck in the near future.

One of the most promising techniques is switching the solvent hydrophobicity, which approach was inspired by switchable solvents.^17^ Bravi and coworkers^18^ have reported the use of a switchable-hydrophilicity eutectic solvent system, based on the previously reported hydrophobic eutectic solvent octanoic acid/dodecanoic acid (3 : 1).^19^ When this solvent is mixed with an aqueous solution of Jeffamine D-230, they form hydrophilic ionic liquid made of the protonated amine and deprotonated acid (forward switching). Then, CO_2_ or acid could be used to protonate back the acid, thus, obtaining the hydrophobic eutectic solvent (backward switching).^18^ With this system, both hydrophilic and hydrophobic biomolecules can be extracted and separated.^18^ However, this approach involved two additional compounds (the amine and carbon dioxide or acid) which later need to be removed further downstream.

In this study, we propose another approach to tune the hydrophobicity of a semi-hydrophobic solvent, consisting of hydrophilic and hydrophobic compounds. With this approach, the eutectic solvent can have a spectrum of hydrophobicity, depending on the composition of the parental compounds. Hence, at the hydrophobic state, the solvent can be used to dissolve hydrophobic solutes, such as lipids. Afterwards, the lipid can be recovered by adding an excess of the hydrophilic parental compound, making the overall solvent hydrophilic. Here in this study, we use sunflower oil and culinary algae oil as model lipids to demonstrate the principle.

To obtain the semi-hydrophobic solvent, a list of compounds was made based on the reported parental compounds for hydrophilic and hydrophobic eutectic solvents, such as C2–C10 carboxylic acids, quaternary ammonium salts (Aliquat® 336 – methyltrioctylammonium chloride and tetrabutylammonium bromide), terpene dl-menthol, and heteroaromatics (imidazole and pyrazole). These non-green compounds are used as a proof-of-principle of this current strategy, which later can be applied on greener compounds with similar or better performance.

Several combinations were made by pairing hydrophobic and hydrophilic compounds, which were screened based on the miscibility with water and dodecane. The combinations which dissolve both water and dodecane were further characterised with polarity estimation and lipids solubility. The polarity of eutectic solvents with different acid/base ratios was estimated by solvatochromic analysis.^14,20–24^ Imidazole/hexanoic acid combination was further used to demonstrate the dissolution and recovery of the model oils.

## Materials and methods

### Materials

The starting compounds of the eutectic solvents used in this study are listed in Table 1. Model oils used in this study were commercial sunflower oil (Jumbo Supermarkten B.V., The Netherlands) and culinary algae oil (Corbion Biotech, Inc., United States). For solvatochromic analysis, *N*,*N*-dimethyl-4-nitroaniline (98+%) was purchased from Alfa Aesar.

**Table 1:** List of chemicals used to prepare eutectic solvents and their properties (unless stated, data taken from PubChem^27^)

Compounds	Supplier, purity	Brønsted or Lewis-	log *K*_ow_	log *S*
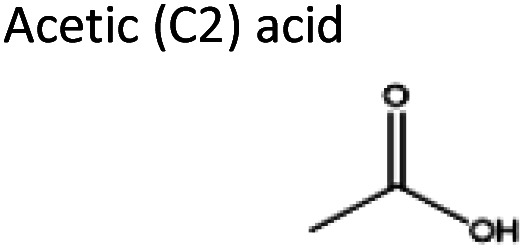	Sigma-Aldrich, 100%	Acid	−0.17	1.22
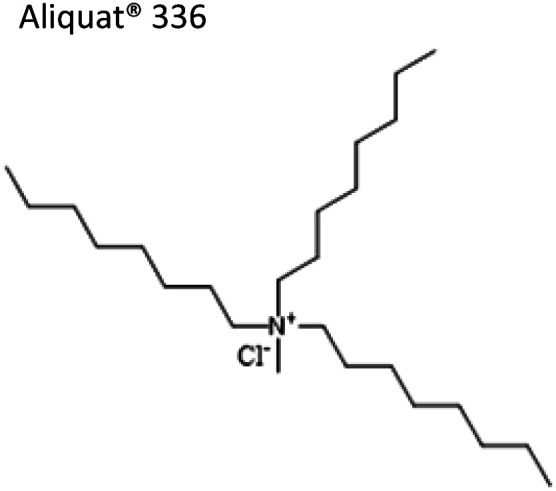	Alfa Aesar, n.a.	Base	6.74^a^	−2.7^b^
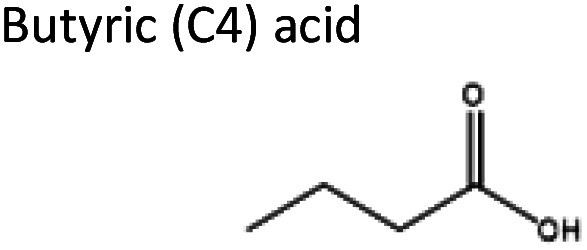	Sigma-Aldrich, ≥99%	Acid	0.79	−0.17
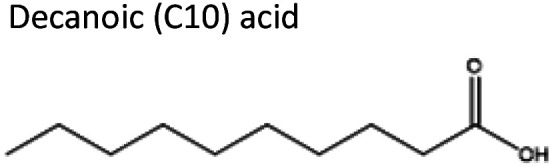	Sigma-Aldrich, ≥98%	Acid	4.09	−3.45
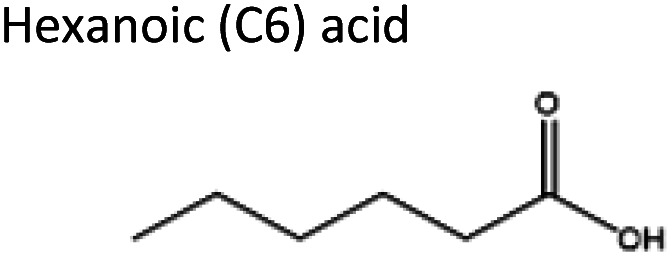	Sigma-Aldrich, ≥99%	Acid	1.92	−1.05
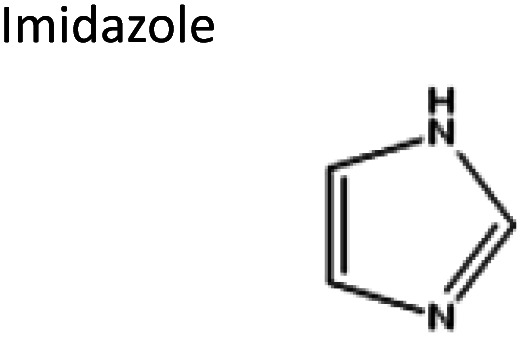	Sigma-Aldrich, ≥99%	Acid or base	−0.08	0.99
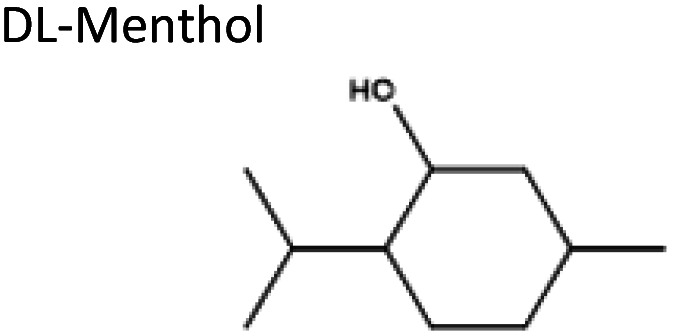	Sigma-Aldrich, ≥98%	Acid or base	3.20	−2.57
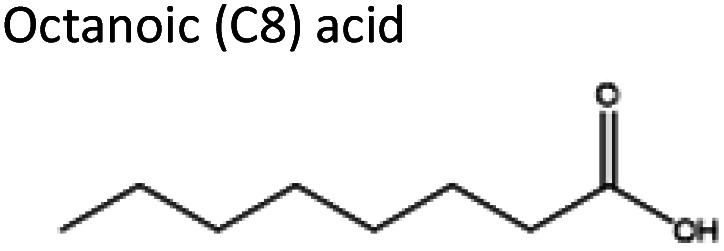	Acros Organics, 99%	Acid	3.05	−2.26
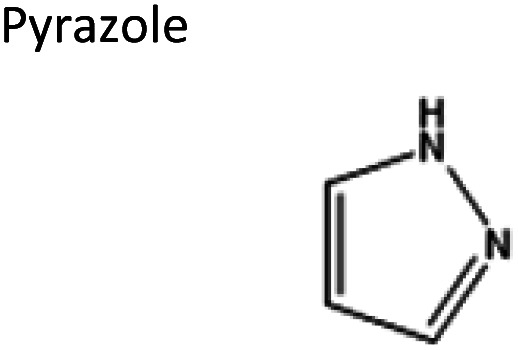	Sigma-Aldrich, ≥99%	Base	0.26	−0.55
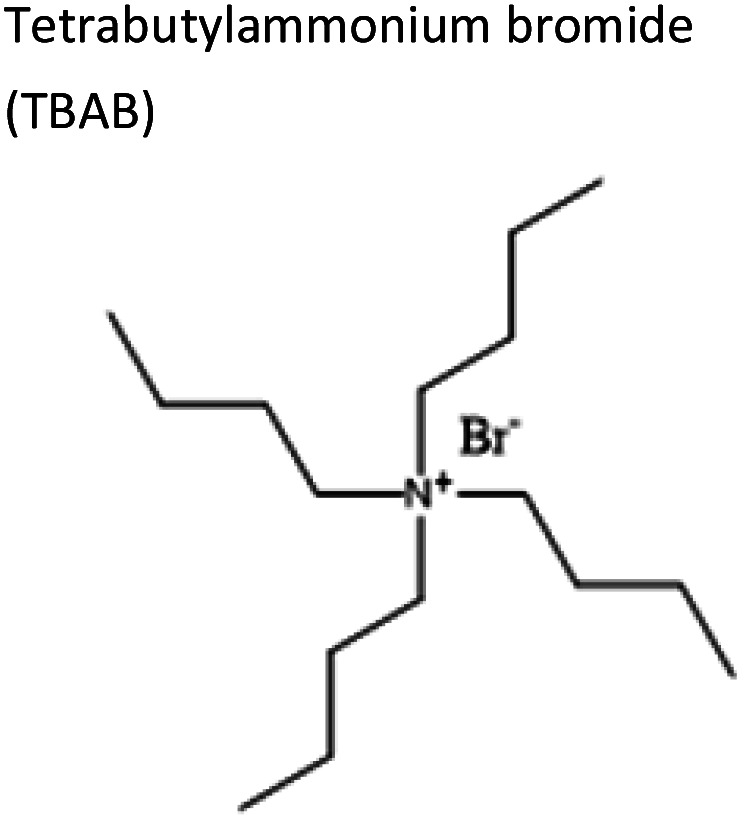	Sigma-Aldrich, ≥99%	Base	1.71^a^	0.27

a Simulated by VCCLAB,^28^http://www.vcclab.org.

b Dissolved in 2 M HCl.^29^

### Preparation of the eutectic solvent

The eutectic solvents were made by mixing the proper combination of acids and bases at 60 °C until clear homogeneous solutions were formed. No prior purification step was performed. The mixtures which did not stay homogeneous for at least 24 hours were categorised as unstable. The stable mixtures were then stored for no longer than one week for further use.

### Dissolution of dodecane and water

The miscibility of dodecane and water in the eutectic solvents were used as indicators of the solvent hydrophobicity. This fast analysis was done by adding dodecane or water into the eutectic mixtures equivolume. Based on the final volume and visual observation of each phase after mixing, the miscibility was evaluated. The combinations which dissolve both dodecane and water were further analysed with solvatochromic analysis.

### Solubility of model oils and water

The solubility of sunflower oil, algae oil, and water were determined in imidazole/hexanoic acid mixture with different molar fraction of imidazole by adding them dropwise until the solution became turbid.

### Recovery of model oils

The recovery of sunflower oil and algae oil from imidazole/hexanoic acid was done by adding a proper amount of imidazole. The addition of imidazole induced a phase split, which was accelerated through centrifugation. The recovered oil formed a top phase, while the hydrophilic mixture together with the remaining oil formed the bottom phase.

The partition coefficient (*K*) and separation efficiency (Eff) of the lipid recovery were calculated by using these equations, respectively:
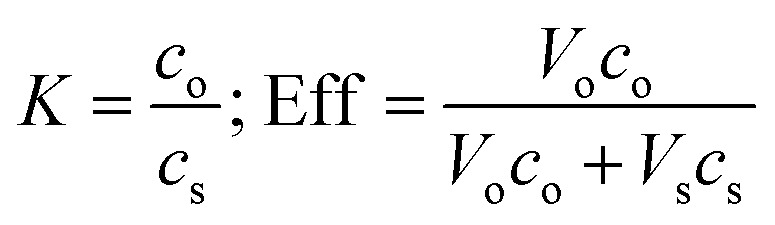
where *c* is the total fatty acids concentration in g g^−1^, *V* is the total amount of the phase in gram, subscript o and s denote the oil-rich and solvent-rich phase, respectively.

### Solvatochromic analysis

The solvatochromic analysis was performed to quantify the polarity of the eutectic solvents that dissolve both dodecane and water. The quantitation was based on Kamlet–Taft dipolarity scale (π*), which is normally based on the red-shift of the absorption spectrum of *N*,*N*-diethyl-4-nitroaniline dissolved in the solvent.^14,20,21^ However, due to the difficulty in finding this dye commercially, the analogue *N*,*N*-dimethyl-4-nitroaniline was used in this study instead.^25,26^ A known amount of the dye was dissolved in the eutectic solvent with a concentration of ∼0.1 mg mL^−1^ before the spectrum measurement using a quartz cuvette with 10 mm light path. The dipolarity scale (π*) was calculated with the formula:
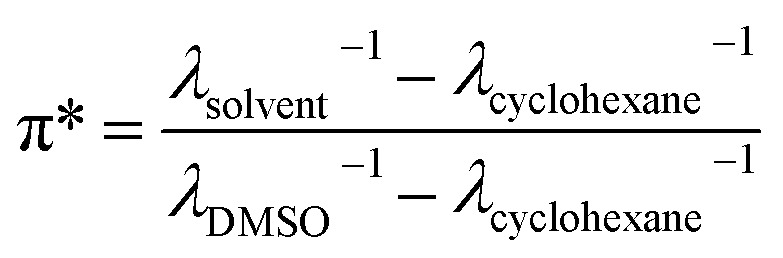
where *λ* is the UV/Vis wavelength [nm] at which maximum absorbance of the dye occurred. The scale uses cyclohexane and dimethyl sulfoxide (DMSO) as references with a value of 0 and 1, respectively.

### Gas chromatography

The analysis of fatty acids was performed using GC-FID system (Agilent Technologies) with H_2_ as a carrier gas. Methylation was performed on the samples (∼5 mg) in advance by adding 3 mL of methanol & 5% H_2_SO_4_ at 100 °C for 1 hour. Then, the methylated products were extracted using hexane that contained C15:0 methyl ester as an internal standard. The samples were then run through Nukol™ column (30 m × 0.53 mm × 1.0 μm, Supelco) with a split ratio of 0.1 : 1 and split flow of 3.55 mL min^−1^. The oven temperature profile was 90 °C to 200 °C at 44.08 °C min^−1^ and held for 7.5 minutes. In this study, the total amount of fatty acids was assumed as the amount of lipid.

## Results and discussion

An outline of the Results and discussion section is given below. First, the potential parental compounds, as listed in Table 1, were combined, and screened to obtain semi-hydrophobic eutectic solvents *via* miscibility with water and dodecane. Then, the polarity of selected mixtures with different composition was quantified by solvatochromic analysis. One mixture with the widest spectrum of dipolarity, imidazole/hexanoic acid, was further selected to demonstrate the lipid recovery *via* changing the solvent composition. Hence, the solubility of model oils was measured in the eutectic solvent with varying imidazole concentration. Furthermore, the actual lipid recovery experiment was performed by inducing phase split. In this part, the partition coefficient, the separation efficiency, and the purity of recovered oils were evaluated. Finally, we compared the basic energy requirement of this process to that of the switchable eutectic solvent approach to evaluate the process feasibility.

### Screening of semi-hydrophobic eutectic solvents

The potential semi-hydrophobic eutectic solvents were screened based on the miscibility with water and dodecane. The miscibility was observed based on the phase volume after being mixed with water or dodecane at 1 : 1 v/v. Each combination was categorised into four groups: (1) dissolve only water, (2) dissolve only dodecane, (3) dissolve both water and dodecane, and (4) dissolve neither water nor dodecane. Additionally, in this study, we used the sum of log *S* as a crude indicator of the mixture hydrophobicity, regardless of the composition. This indicator may not properly function for other combinations outside this study. It is also worth to mention that this indicator does not imply any thermodynamic properties of the mixtures.

Table 2 shows the combinations of each group. As expected, combinations which belong to Group 1 are made from hydrophilic acids and bases (sum log *S* > 0.8), whereas Group 2 are generally combinations of two hydrophobic compounds (sum log *S* < −2.8). Furthermore, all combinations of Aliquat® 336 always belong to Group 2 due to the long alkyl chains of the cation. Semi-hydrophobic eutectic solvents, Group 3, are combinations of hydrophilic acids and hydrophobic bases, or *vice versa* (−2.8 < sum log *S* < 0.8). Moreover, despite the similar values of sum log *S* to Group 3, Group 4 showed little to no miscibility with water and dodecane.

**Table 2:** Combinations of characterised deep eutectic solvents in this study. The group categories are (1) dissolve only water, (2) dissolve only dodecane, (3) dissolve both water and dodecane, and (4) dissolve neither water nor dodecane

Group	Base	Acid	Sum log *S*	Base : acid ratio
2	Aliquat® 336	Decanoic acid	−6.15	1 : 2
2	dl-Menthol	Decanoic acid	−6.02	1 : 2
2	Aliquat® 336	dl-Menthol	−5.27	1 : 2
2	Aliquat® 336	Octanoic acid	−4.96	1 : 2
2	dl-Menthol	Octanoic acid	−4.83	1 : 2
2	Pyrazole	Decanoic acid	−4.00	1 : 2
2	Aliquat® 336	Hexanoic acid	−3.75	1 : 2
2	dl-Menthol	Hexanoic acid	−3.62	1 : 2
Unstable	TBAB	Decanoic acid	−3.18	1 : 2
2	dl-Menthol	Pyrazole	−3.12	3 : 1
2	Aliquat® 336	Butyric acid	−2.87	1 : 2
2	Pyrazole	Octanoic acid	−2.81	1 : 2
2	dl-Menthol	Butyric acid	−2.74	1 : 2
Solid	Imidazole	Decanoic acid	−2.46	1 : 2
3	TBAB	dl-Menthol	−2.30	1 : 3
4	TBAB	Octanoic acid	−1.99	1 : 2
4	Aliquat® 336	Imidazole	−1.71	1 : 1
3	Pyrazole	Hexanoic acid	−1.60	1 : 2
Unstable	dl-Menthol	Imidazole	−1.58	3 : 1
2	Aliquat® 336	Acetic acid	−1.48	1 : 2
3	dl-Menthol	Acetic acid	−1.35	1 : 2
3	Imidazole	Octanoic acid	−1.27	1 : 2
4	TBAB	Hexanoic acid	−0.78	1 : 2
3	Pyrazole	Butyric acid	−0.72	1 : 2
3	Imidazole	Hexanoic acid	−0.06	1 : 2
1	TBAB	Butyric acid	0.10	1 : 2
Solid	Imidazole	Pyrazole	0.44	1 : 1
3	Pyrazole	Acetic acid	0.67	1 : 2
1	Imidazole	Butyric acid	0.82	1 : 2
1	TBAB	Imidazole	1.26	1 : 2
1	TBAB	Acetic acid	1.49	1 : 2
1	Imidazole	Acetic acid	2.21	1 : 2

Besides that, there were also unstable combinations, such as TBAB/decanoic acid and dl-menthol/imidazole, which formed solids when mixed with water. The solid formation indicated the alteration of solid–liquid equilibrium of the eutectic combination. This alteration may be due to either the incorporation of water in the overall supramolecular interaction or the change in the eutectic mixture composition. Based on reported previous studies, the presence of water in choline chloride/urea caused a further depression of melting point.^30,31^ Therefore, it is more likely that the latter took place. Due to the large difference in water affinity of the constituents, the hydrophilic TBAB and imidazole leached to water. Hence, the effective molar ratio of TBAB or imidazole in the non-aqueous phase decreased, resulting in less reduction of the melting point and the solid formation. This leaching phenomenon may also occur in other combinations, but with less obvious visual effect. In fact, several cases of leaching have been reported in the mixtures based on tetrabutylammonium chloride, and dl-menthol when combined with short carboxylic acid.^32^

### Solvatochromic analysis of semi-hydrophobic eutectic solvent

**Fig. 1:**
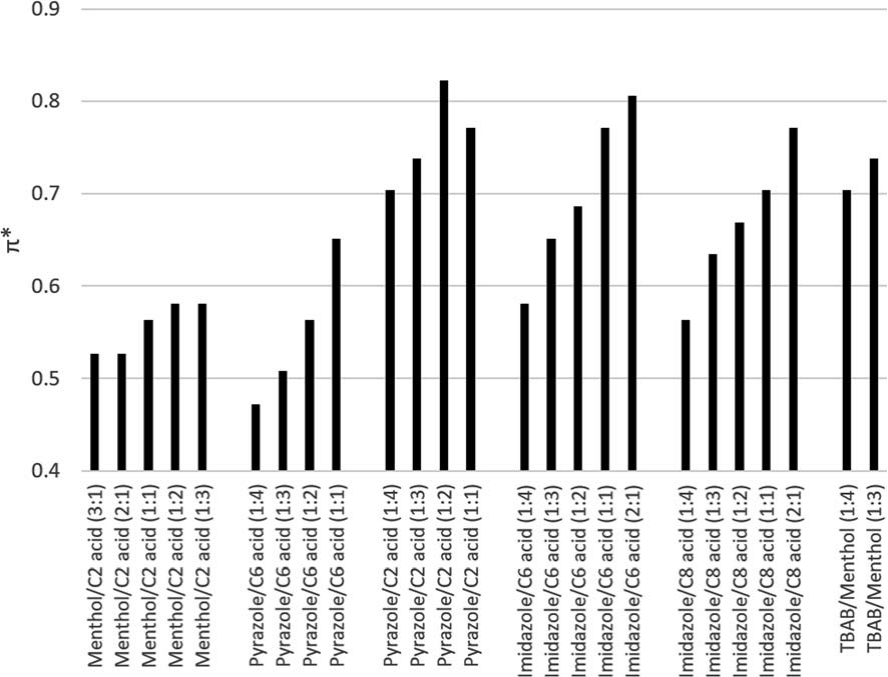
Dipolarity π* values of selected semi-hydrophobic eutectic solvents. Imidazole/hexanoic acid showed the largest change of dipolarity.

The solvatochromic analysis was performed on the eutectic solvents in Group 3 to measure the solvent polarity with various compositions. Fig. 1 shows the polarity scale of selected eutectic solvents with various compositions. In general, the polarity is heavily influenced by the nature of the parental compounds, with stronger emphasis from the bases. The permanent charge of TBAB caused a high dipolarity value, followed by the aromatic imidazole and pyrazole, and lastly by dl-menthol, which is neutral. Besides that, the acids also influence the mixture polarity to a lesser degree. For example, pyrazole/acetic acid has higher dipolarity than pyrazole/hexanoic acid. Similarly, it can also be observed between imidazole/hexanoic acid pair and imidazole/octanoic acid to a lower extent. These findings agree with the previously reported results.^14,20,21^

More importantly, this result confirms our hypothesis that the polarity of these solvents is influenced by the composition of parental compounds. For example, in the case of imidazole/hexanoic acid and pyrazole/hexanoic acid, the solvent polarity increases at lower concentrations of the hydrophobic acid. Hence, the solvent hydrophobicity can be tuned by changing the constituent molar ratio, which is the basic principle of polarity shifting proposed in this study. Among the combinations, imidazole/hexanoic acid showed the largest change in dipolarity value (Fig. 1), indicating the widest range for tuning the hydrophobicity. Hence, this combination was used to demonstrate the lipid recovery by this hydrophobicity shifting approach.

### Solubility of model oils and water in imidazole/hexanoic acid eutectic solvent

**Fig. 2:**
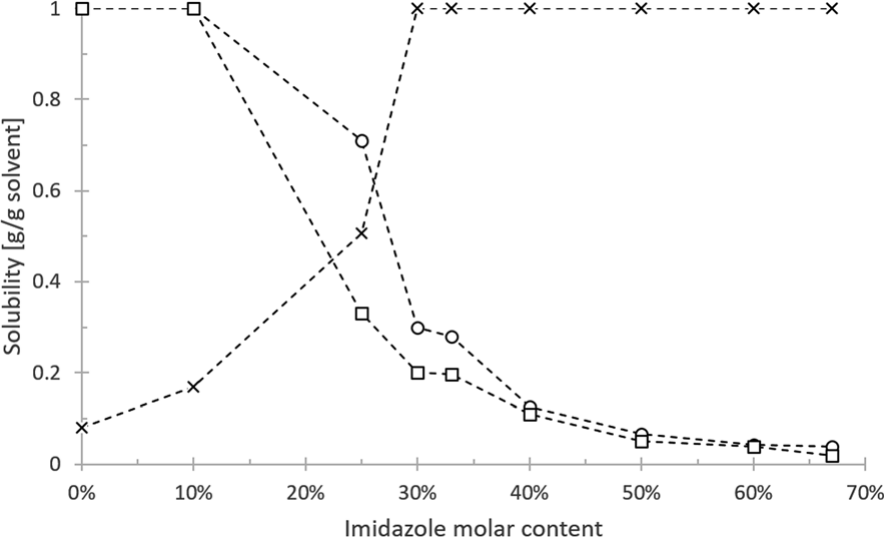
Solubility of water (cross), sunflower oil (circle), and algae oil (square) in imidazole/hexanoic acid with different molar fraction of imidazole.

The eutectic solvent imidazole/hexanoic acid was further characterised by measuring the solubility of sunflower oil, algae oil, and water in different molar ratio (Fig. 2). Since imidazole is a polar and hydrophilic compound, the increased presence of imidazole results in the higher degree of solvent hydrophilicity. Thus, the model oils dissolved well in the solvent with low imidazole content and became less soluble at higher imidazole molar ratio. Sunflower oil generally dissolves better than algae oil in the eutectic solvent. This solubility difference may be associated with the different chemical composition of the oils (Table S1†). On the other hand, the solubility of water is positively correlated to imidazole content and reaches complete miscibility at 30%.

Despite hexanoic acid being the dominant constituent, the solvent still has a high affinity towards water at >30% imidazole molar content. This might be explained by the result of acid–base interaction between hexanoic acid and imidazole (p*K*_a_ = 4.88 and p*K*_b_ = 7.05, for respective compounds). Unlike the normally reported eutectic solvents that formed due to hydrogen bonding,^2,11,33,34^ this interaction produces the ionic liquid imidazolium hexanoate.^35^ The formed ions could interact strongly with water, causing the high affinity towards water. Furthermore, the presence of the permanent charge of imidazolium hexanoate can also explain the rapid decline of lipid solubility in the range of 10–30% imidazole molar content. Assuming the spontaneous formation of imidazolium hexanoate at equimolar (1 : 1) ratio, the mixture at other ratios might very well be composed of imidazolium hexanoate and the unreacted species. For example, at imidazole/hexanoic acid (1 : 3), which equivalent to 25% imidazole content, all imidazole reacted with hexanoic acid to form imidazolium hexanoate and unreacted hexanoic acid with 1 : 2 ratio. The melting point of the imidazole and hexanoic acid combination at different ratios showed eutectic behaviour, *i.e.*, depressed melting point (phase diagram: Fig. S1†).

### Recovery of model oils by phase split

**Fig. 3:**
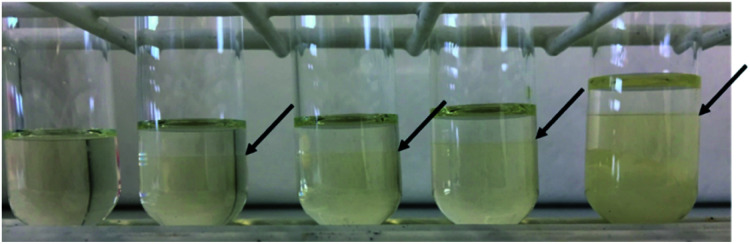
Recovery of sunflower oil from eutectic solvent *via* induced phase split by adding imidazole. The arrows show the interface between the recovered oil and modified eutectic solvent. From left to right, the eutectic solvent contained 25%, 40%, 50%, 60%, and 75% imidazole molar content. The initial concentration of sunflower oil is 0.55 g g^−1^ solvent.

The proposed strategy to shift the solvent hydrophobicity by adding imidazole was then applied for the recovery of the model oils. This experiment began with the model oils dissolved in imidazole/hexanoic acid (1 : 3) – 25% imidazole molar content, resulting in a homogeneous solution (Fig. 3). The initial concentration of sunflower oil and algae oil in the eutectic solvent is 0.55 and 0.28 g oil/g solvent, respectively. Then, excess of imidazole was added to the system until reaching 40%, 50%, 60%, and 75% imidazole content. The high imidazole concentrations induced phase split between the light lipid-rich phase and the heavy solvent-rich phase (the interface is shown by the arrows). At 75% imidazole molar content (3 : 1), not all imidazole dissolved and remained as white solids in the bottom part of the tube. This indicated that this system was beyond saturation (Fig. S1†). All formed phases were then analysed by gas chromatography to measure the total fatty acid content, which further assumed to be the lipid content. Based on this analysis, partition coefficient, recovery efficiency, and purity of the recovered product were evaluated. The overview can be seen in Table 3.

**Table 3:** Results overview of the lipid recovery experiment. *c*_o_ and *c*_s_ are the lipid concentration in oil- and solvent-rich phase, respectively; *V*_o_ and *V*_s_ are the total mass of oil- and solvent-rich phase, respectively, *K* is the lipid partition coefficient 
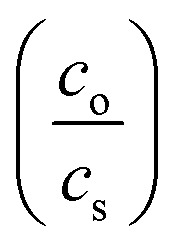
, and Eff is the separation efficiency

Imidazole [mol%]	*c*_o_ [g g^−1^]	*V*_o_ [g]	*c*_s_ [g g^−1^]	*V*_s_ [g]	*K*	Eff [%]
**Sunflower oil**						
40%	0.98 ± 0.03	0.301 ± 0.017	0.13 ± 0.00	1.406 ± 0.001	7.3 ± 0.1	61
50%	0.90 ± 0.12	0.375 ± 0.012	0.08 ± 0.00	1.495 ± 0.027	11.0 ± 1.4	73
60%	0.95 ± 0.00	0.384 ± 0.000	0.14 ± 0.00	1.737 ± 0.000	6.8 ± 0.0	60
75%	1.01 ± 0.00	0.369 ± 0.013	0.13 ± 0.07	2.495 ± 0.043	9.5 ± 5.3	56

**Algae oil**						
40%	0.75 ± 0.11	0.110 ± 0.011	0.11 ± 0.05	1.296 ± 0.009	7.9 ± 4.5	38
50%	0.82 ± 0.07	0.176 ± 0.005	0.05 ± 0.01	1.413 ± 0.012	17.0 ± 4.4	67
60%	0.85 ± 0.01	0.183 ± 0.042	0.03 ± 0.01	1.627 ± 0.022	27.7 ± 6.6	75
75%	0.86 ± 0.02	0.206 ± 0.017	0.03 ± 0.01	2.352 ± 0.001	27.9 ± 6.3	71

#### Partition coefficient

**Fig. 4:**
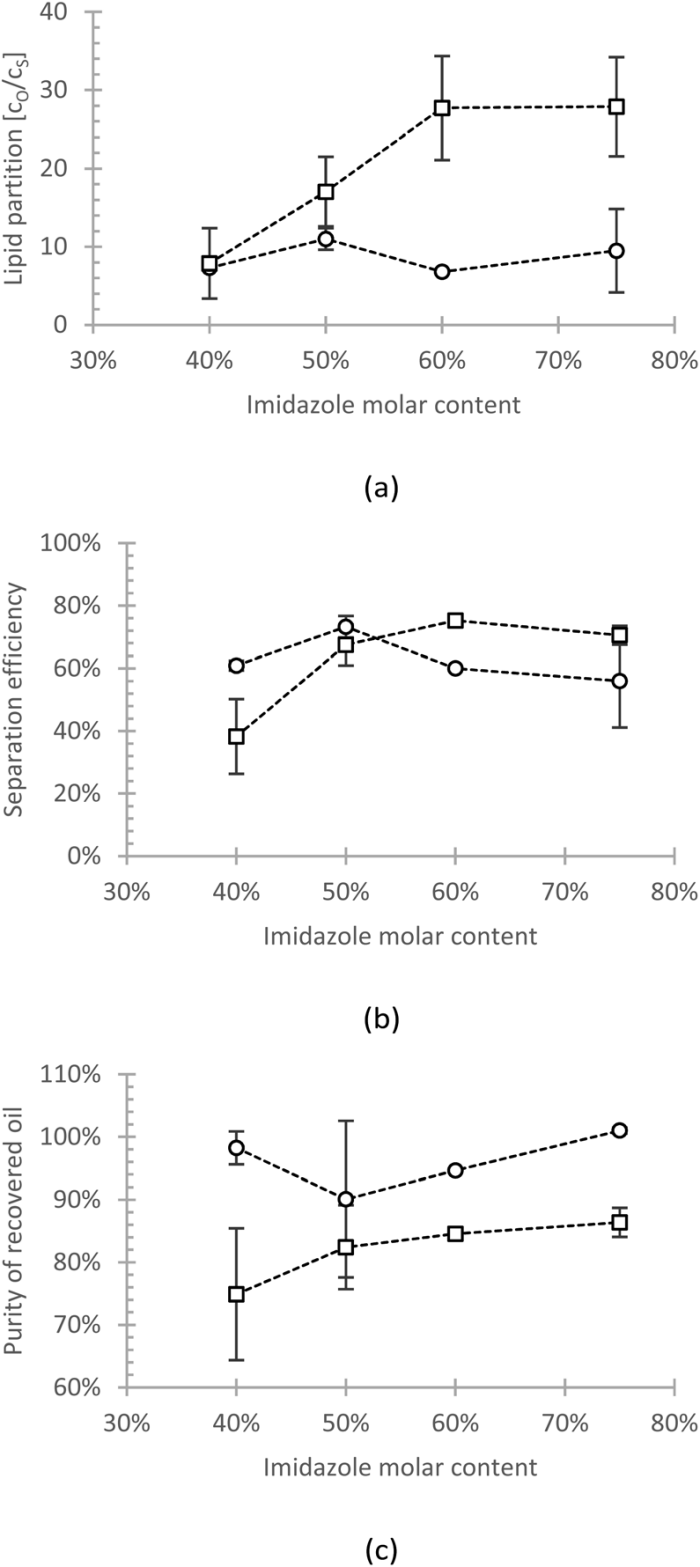
(a) The partition coefficient, (b) the recovery efficiency, and (c) the purity of the recovered sunflower oil (circle) and algae oil (square) from imidazole/hexanoic acid by adding imidazole.

The partition coefficient is important to understand how the lipid distributed in the light and heavy phase. With higher partition coefficients, the lipids are less distributed and more likely to be found in the light phase. Fig. 4a shows the partition of the oils between the light and heavy phase, based on the concentration ratio. The partition of algae oil is positively correlated to imidazole content, whereas that of sunflower oil remains relatively constant. Moreover, generally, algae oil reached higher partitions than sunflower oil. The highest obtained partition coefficient for sunflower and algae oil is 11 and 28, respectively.

The different behaviour may be owed to the different nature of the two oils, *e.g.*, the difference in fatty acid profile (Table S1†). The algae oil contains >93% monounsaturated fatty acid (MUFA) C18:1, whereas the most abundant fatty acid in sunflower oil is polyunsaturated fatty acid (PUFA) C18:2 (58%). It is known that the unsaturated bonds in fatty acids can interact with cations or other aromatic compounds.^36–38^ Hence, the polar solvent may have a relatively higher affinity towards PUFA than towards MUFA. This causes the relatively better dissolution of sunflower oil than algae oil in the polar state of eutectic solvent, which leads to a lower partition coefficient of sunflower oil.

#### Recovery efficiency

Besides the partition behaviour, we also evaluated the recovery efficiency of the model oils, which is based on the oil mass. Hence, the recovery is not only determined by the partition equilibrium, but also by the amount of the formed phases. Fig. 4b shows the recovery efficiency of the model oils, which is influenced by the imidazole content. For sunflower oil, the maximum recovery of 73% was achieved at 50% imidazole content, and it tends to decline at higher imidazole content despite being not statistically significant. Whereas for algae oil, the recovery resembles the trend of partitioning, which rises with higher amount of imidazole. The highest achieved efficiency for algae oil was 75% at 60% imidazole molar content.

As mentioned before, the recovery is determined by the amount of the phase formed. The added imidazole, which induced the phase split, in fact, created a dilution effect on the heavy solvent phase. This dilution then reduces the recovery efficiency since, with the increased amount of bottom phase, the amount of unrecovered lipid is also increased. This may explain the declining trend of sunflower oil at high imidazole content. The slightly declining trend can also be observed in the case of algae oil. However, since less algae oil is dissolved in the solvent-rich phase when compared to sunflower oil (>2.5-fold in partition coefficient), the dilution effect is not as obvious.

#### Purity of recovered oil

Furthermore, the concentration of total fatty acid in the oil-rich phase was further assumed to represent the purity of recovered oil (Fig. 4c). The overall purity of the recovered oils was relatively high, with sunflower oil reached >90%, and algae oil reached 75–86%. The possible impurities in the oil-rich phase include eutectic solvent constituents, hexanoic acid, imidazole, and their derivatives. Based on the gas chromatography analysis, the presence of hexanoic acid is in the range of 2–5%, which decreases with higher imidazole content. Besides that, the remaining mass would be associated with several possible compounds, such as imidazole, glycerol, the polar functional groups of polar lipids, and vitamins.

### Eutectic solvent regeneration

Despite the little energy requirement for the lipid recovery, the regeneration of the eutectic solvent to its initial ratio is not yet considered. One can simply add hexanoic acid to readjust the molar composition, but that approach is not sustainable since the eutectic solvent would accumulate along with the process cycles, while fresh imidazole and hexanoic acid need to be continuously supplied as makeup. Therefore, other techniques for the eutectic solvent regeneration are still being explored in the future studies.

## Conclusion

In this study, a new approach of lipid recovery from a semi-hydrophobic eutectic solvent was developed by shifting the hydrophobicity spectrum. The tuneable hydrophobicity of the eutectic solvent was achieved by changing the compositional ratio. A solvent mixture which is composed of imidazole and hexanoic acid was shown to have this tuneable hydrophobicity, which hydrophobicity reduced with higher imidazole. Therefore, this combination was used to demonstrate the dissolution and recovery of sunflower and algae oil. By adding imidazole, the solubility of the model oils decreased, which induced a phase split between the upper oil-rich phase and the lower solvent-rich phase. With this approach, about 75% of the oils can be recovered with relatively high purity (which can reach >90%). The recovery was also affected by the natural composition of the model lipids.

## Conflicts of interest

The authors declare no conflict.

## Supplementary Material

RA-011-D1RA00306B-s001
