# Supplementary material for: Eutectic solvents with tuneable hydrophobicity: lipid dissolution and recovery
Source: RSC Adv. 2021 Feb 19;11(14):8142–9. doi: 10.1039/d1ra00306b (PMC8695114; doi:10.1039/d1ra00306b)
Supplement: RA-011-D1RA00306B-s001 [file RA-011-D1RA00306B-s001.pdf]

Electronic Supplementary Material (ESI) for RSC Advances.
This journal is © The Royal Society of Chemistry 2021

Eutectic solvents with tuneable hydrophobicity: lipid dissolution and recovery

Calvin Lo,*[a] Jeltzlin Semerel,[a] Corjan van den Berg,[a] René H. Wijffels [a,b] and Michel H. M. Eppink [a]

Electronic Supplementary Information

Phase diagram of imidazole/hexanoic acid

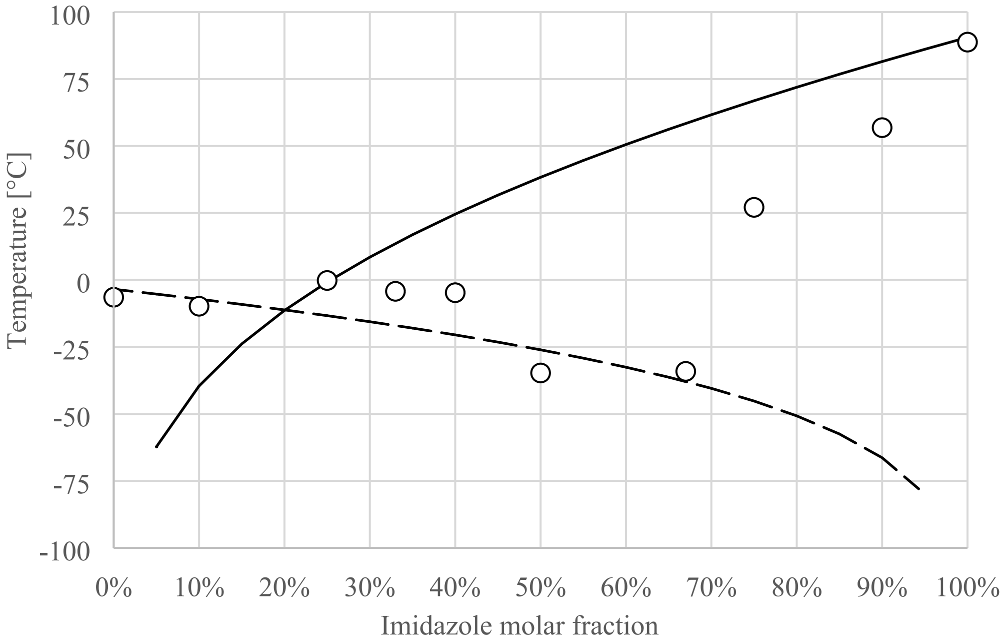

Figure S1. Phase diagram of imidazole/hexanoic acid eutectic solvent. The circles represent the DSC onset temperatures; the continued and dashed line represent the model of ideal eutectic behaviour of imidazole and hexanoic acid, respectively.

The melting temperatures were the onset temperatures measured by differential scanning calorimeter (Perkin-Elmer DSC 8000 series). The temperature profile was as follow: 1) fast heating from 20 °C to 90 °C (100 °C /min), 2) hold at 90 °C for 10 min, 3) fast cooling from 90 °C to -90 °C (100 °C /min), 4) scanning from -90 °C to 90 °C (10 °C /min), 5) fast cooling from 90 °C to -90 °C (100 °C /min), 6) scanning from -90 °C to 90 °C (10 °C /min), 7) fast cooling to 20 °C.

The ideal eutectic behaviour was calculated with this equation:

$$\ln\left(x_i\right) = \frac{\Delta_m H}{R}\left(\frac{1}{T_m} - \frac{1}{T}\right)$$

where x_i is the liquid molar fraction of compound i, T is the apparent temperature [K], T_m and $\Delta_m H$ are the melting temperature and enthalpy of the pure compound i, respectively, and R is the universal gas constant. The values of T_m and $\Delta_m H$ for imidazole are 363.7 K and 12.5 kJ/mol, respectively (Almeida & Monte, 2012); and those for hexanoic acid are 269.7 K and 16.98 kJ/mol, respectively (https://www.chemeo.com/cid/49-496-8/Hexanoic%20acid).

Fatty acid composition of sunflower oil and algae oil

Table S1. The fatty acid profile of the model oils used in this study

**Fatty Acid [%]**	Sunflower oil	Algae oil
C8:0	0.00%	0.00%
C10:0	0.00%	0.00%
C12:0	0.00%	0.14%
C13:0	0.00%	0.00%
C14:0	0.10%	0.47%
C14:1 cis-9	0.00%	0.00%
C16:0	6.85%	1.77%
C16:1	0.17%	0.30%
C16:2	0.00%	0.00%
C16:3	0.00%	0.00%
C17:0	0.00%	0.00%
C18:0	3.16%	0.99%
C18:1	30.70%	93.35%
C18:2	57.86%	1.69%
C18:3	0.00%	0.00%
C19:0	0.09%	0.19%
C20:0	0.24%	0.04%
C20:1	0.17%	1.07%
C20:2	0.00%	0.00%
C20:3	0.00%	0.00%
C20:4	0.00%	0.00%
C20:5	0.00%	0.00%
C22:0	0.65%	0.00%
C22:1	0.00%	0.00%
C24:0	0.00%	0.00%
C22:6	0.00%	0.00%

Reference

Almeida, A. R. R. P., & Monte, M. J. S. (2012). Thermodynamic study of phase transitions of imidazoles and 1-methylimidazoles. *Journal of Chemical Thermodynamics*, *44*(1), 163–168. https://doi.org/10.1016/j.jct.2011.08.017
